# Prevalence of persons contacting general practice for psychological stress in Denmark

**DOI:** 10.1080/02813432.2018.1499494

**Published:** 2018-09-03

**Authors:** Jesper Lykkegaard, Marianne Rosendal, Karen Brask, Lars Brandt, Anders Prior

**Affiliations:** a Research Unit of General Practice, Department of Public Health, University of Southern Denmark, Odense, Denmark;; b Department of Occupational and Environmental Medicine, OUH Odense University Hospital, Odense, Denmark;; c Research Unit for General Practice, Department of Public Health, Aarhus University, Aarhus, Denmark

**Keywords:** stress, psychological, primary health care, family practice, general practice, prevalence, clinical audit, public health

## Abstract

**Objective:** The prevalence of psychological stress has previously been estimated based on self-reported questionnaires. This study aimed to investigate the prevalence of persons who contact the general practitioner (GP) for psychological stress and to explore associations between psychological stress and characteristics relating to the patient, the GP, and area-specific socioeconomic factors.

**Design:** Cross-sectional computer assisted journal audit.

**Setting:** General practice in the Region of Southern Denmark.

**Subjects:** Patients aged 18–65 years with a consultation during a six-month period that was classified with a stress-related diagnosis code.

**Main outcome measures:** Six months prevalence of GP-assessed psychological stress and characteristics relating to the patient, the GP, and area-specific socioeconomic factors.

**Results:** Fifty-six GPs (7% of the invited) identified 1066 patients considered to have psychological stress among 51,422 listed patients. Accordingly, a 2.1% six months prevalence of psychological stress was estimated; 69% of cases were women. High prevalence of psychological stress was associated with female sex, age 35–54 years, high education level and low population density in the municipality, but not with unemployment in the municipality or household income in the postal district. GP female sex and age <50 years, few GPs in the practice and few patients per GP were also associated with a higher prevalence of psychological stress.

**Conclusions:** A total of 2% of the working-age population contacted the GP during a six-month period for psychological stress. The prevalence of psychological stress varies with age, sex and characteristics of both the regional area and the GP.

**Key points**
  Psychological stress is a leading cause of days on sick leave, but its prevalence has been based on population surveys rather than on assessment by health care professionals.
  • This study found that during six months 2.1% of all working-age persons have at least one contact with the GP regarding psychological stress.
  • The six months prevalence of psychological stress was associated with patient age and sex, GP age and sex, practices’ number of GPs and patients per GP, and area education and urbanization level.

## Introduction

Psychological stress is a common condition and most likely a frequent reason for consulting the general practitioner (GP) [1]. In western countries, it is reported as one of the conditions causing the highest number of days on sick leave [2–5]. The severity of the condition also mirrors in its association with adverse outcomes such as development of chronic diseases [6–8] and increased mortality [9]. Nevertheless, there is a general lack of studies investigating the prevalence of persons in the general population who contacts the healthcare systems with symptoms of psychological stress.

Stress was first described by Hans Selye in 1936 [10]. It is a condition characterized by cognitive, practical and social disabilities and is considered to be an adverse psycho-physiological reaction to challenges that the patient has difficulties coping with. Psychological stress is currently not classified as an independent disease with a separate diagnostic code, but psychological stress symptoms are included in the criteria for various psychiatric diagnoses. Consequently, doctors use various diagnoses to describe the condition when patients visit the healthcare system for psychological stress. The condition is primarily evaluated based on the patient´s experience of symptoms [11] and how this is communicated to and interpreted by the medical doctor. There is no clinically validated biomedical or psychometric test to measure psychological stress. Previous studies on the prevalence of psychological stress have mostly been conducted as population surveys based on self-reported symptoms rather than on assessments by health care professionals [12].

Danish general practitioners (GPs) are generally experienced in evaluating patients with psychological stress. Severe psychological stress often results in sick leave, in which case the patient needs a sick note from the GP. The GPs act as gatekeepers to the rest of the healthcare system, and stress-related healthcare services from a psychologist or a psychiatrist require referral from the GP. Furthermore, the variety of physical and mental symptoms associated with psychological stress makes the patient likely to see the GP [13,14].

This study aimed to investigate the prevalence of persons who contact the GP for psychological stress and to explore associations between psychological stress and characteristics relating to patient, GP and area-specific socioeconomic factors.

## Material and methods

### Design

All GPs in the Region of Southern Denmark were invited to a one-day seminar on psychological stress. As a mandatory preparation, the GPs were asked to identify all patients assessed to have psychological stress during a consultation in the six-month period from 1 October 2015 to 31 March 2016. For each patient identified, the GPs filled in a registration sheet. Participating GPs were paid for two hours’ work for recording the data and partially reimbursed for absence from their clinic during the seminar.

### Setting

Denmark has 5.7 million citizens. The Danish healthcare system is virtually free of charge and includes free access to GP services. Free psychiatric services and partial remuneration for psychological services require a referral from the GP. GPs are private entrepreneurs working under a contract with the Danish Regions. About 98% of the Danish population is listed with a general practice. On average, there are 1.7 GPs per general practice. All Danish GPs use electronic medical records (EMRs). The majority of GPs classify each problem in the consultation using the International Classification of Primary Care (ICPC) [15].

### Identification of patients

When signing up for the project, the GPs were required to tell which EMR system they used. Accordingly, the GPs were provided a specific procedure to follow in order to identify patients with possible psychological stress. The procedure identified all patients aged 18–65 years with a consultation in the six-month period that was coded with psychological symptoms or a diagnosis that could be considered as psychological stress. The age group was chosen based on the age of majority (18 years) and retirement (65 years) in Denmark. The GPs were asked to go through the list of retrieved patients and select those considered to have psychological stress based on information in the EMR and any further knowledge that the GP had about the patient. The GPs were provided with a list of physical, cognitive and behavioural symptoms associated with stress (Supplementary file), but the identification was intentionally primarily based on the GPs’ gut feeling and expert opinion. For each patient identified with stress, a registration sheet was filled in.

The ICPC codes used for the search in all EMRs were: P01, P02, P03, P06, P29, P74, P76, P99 and Z05. Specific psychiatric disorders, except for anxiety, depression and “non-specified”, were not included (see full list of diagnostic codes and labels in the Supplementary material).

### Data

The design of the registration sheet has previously been used by Audit Project Odense in multiple audits [16]. The GPs recorded the patient’s age and sex and ticked whether the patient was diagnosed with any psychiatric disorder. In a separate questionnaire, the GPs reported basic characteristics about themselves and their clinic including the number of listed patients (see the registration sheets in the Supplementary material). The postal addresses of the GP clinics were used to identify the municipality and the postcodes, which were used to retrieve average annual household incomes (dichotomized at 60,000€), unemployment rates (dichotomized at 3%), education levels (dichotomized at 20% of citizens having more than 12 years of education) and population densities (dichotomized at 75% of citizens living in an urbanised area) from the Danish Ministry for Economic Affairs and the Interior [17]. Each cut point for dichotomization was chosen based on the distribution of the variable in the study dataset. The goal was to separate areas and patients in two equally sized groups with maximal difference regarding the variable (Table 2).

### Analyses and statistics

The age and sex composition of listed patients in each GP clinic was assumed to be similar to the composition in the municipality, where the clinic was located. The number of listed female and male patients within each age group was estimated on this basis. The prevalence of psychological stress was calculated by dividing the number of identified cases with the sum of listed patients in the working age. To test the robustness of the estimates and to investigate risk factors for psychological stress, the prevalence was also estimated in stratified groupings of the population according to the characteristics of patient, GP and area-specific socioeconomic factors. Logistic regression was used to estimate adjusted odds ratios (OR) for psychological stress using several adjustment models. Robust variance estimation of the 95% confidence intervals (CI) was used to account for patient clustering by GPs. Model 1 included each variable alone. Model 2 included GP characteristics. Model 3 included area-specific socioeconomic characteristics, and Model 4 included both GP and area-specific socioeconomic characteristics.

GPs who participated as the only GP from a clinic with several GPs may have had more patients with psychological stress, which could bias towards a higher prevalence estimate. Thus, separate prevalence estimates were made for clinics where all GPs participated in the audit.

All analyses were performed in Stata 14.1 (StataCorp, College Station, TX, USA).

### Ethics

The study was strictly observational. All participating GPs volunteered. For quality improvement purposes, Danish healthcare professionals are allowed to look back six months in their patient records without any permission from the ethical committee or the Danish Data Protection Agency. No individuals could be identified in the study’s data that was delivered by the GPs.

## Results

Invitations were mailed to all 807 GPs in the Region of Southern Denmark, and 59 GPs (7.3%) from 34 practices (9.3%) participated in the audit. Participating GPs were representative for the region (Table 1). One practice with three GPs was excluded as an outlier because it identified more than twice as many patients as the practice with the second highest prevalence of psychological stress.

**Table 1.** Characteristics of participating GPs compared to the total region.

	Participating GPs	All GPs in the region^a^
Number of GPs	56	807
Number of clinics	33	365
Average number of listed patients per GP	1509	1501
Proportion of female GPs	61%	49%
Average age of GPs	50 years	52 years
Proportion in single-handed clinics	14%	18%
Proportion in clinics >2 GPs	70%	60%

a Source: The Region of Southern Denmark, Autumn 2015.

**Figure 1.**
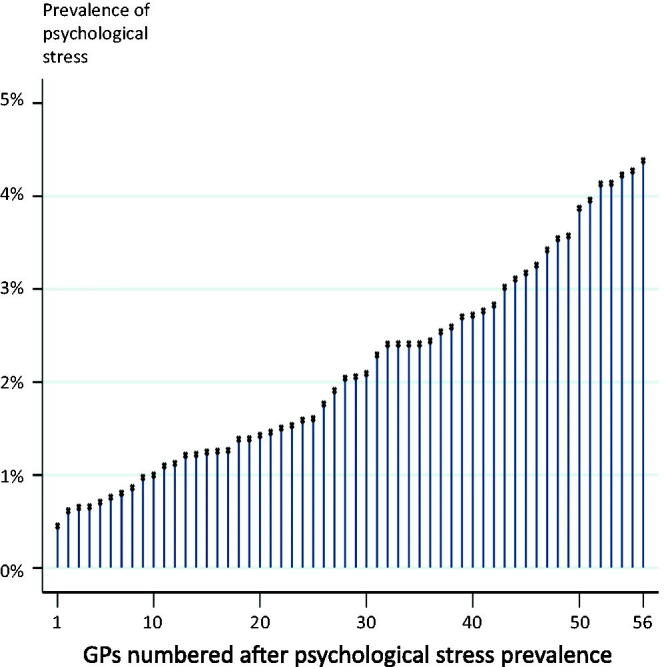
Variation among GPs in listed patients' prevalence of psychological stress.

The variation in the prevalence of psychological stress was considerable among the GPs, ranging from 0.5% to 4.4% (Figure 1).

**Figure 2.**
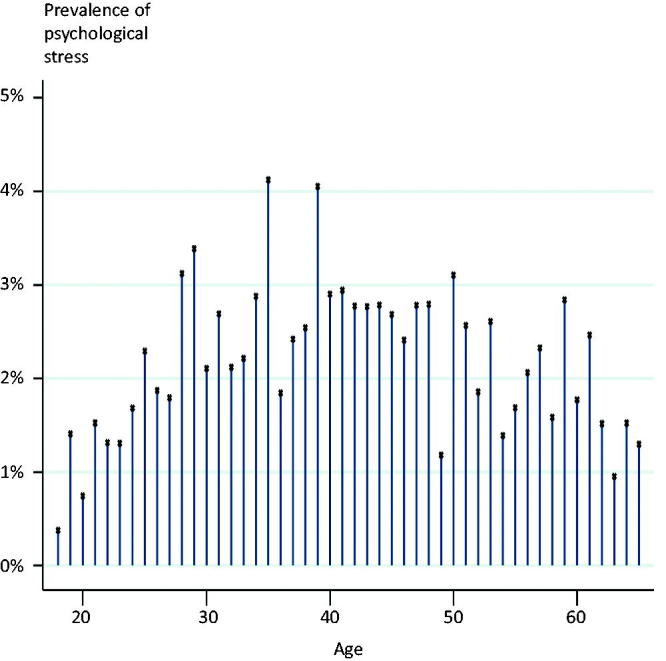
Age distribution of psychological stress.

The EMR search procedure identified 4213 patients who had a consultation with one of the chosen diagnosis codes (supplementary material) during the six months. From these patients, the GPs assessed 1066 patients to have psychological stress. Based on the reported list sizes, the participating GPs had 51,422 listed patients aged 18–65 years (7.0% of the working-age population in the region). Thus, the estimated total prevalence of psychological stress as assessed by the GPs was 2.1% (Table 2). The prevalence was highest in patients aged 35-54 years (Figure 2).

**Table 2.** Prevalence of psychological stress according to the characteristics of patients, GPs, practices and geographic areas.

Characteristics		Number (%)	Unit	n/N patients	Prevalence (%)
Total prevalence		1066 (100)	Patients	1066 / 51.422	2.1
Patient sex^a^	Female	739 (68)	Patients	739 / 25.306	2.9
	Male	325 (32)	Patients	325 / 26.116	1.2
Patient age	18–34 years	292 (27)	Patients	292 / 16.744	1.7
	35–54 years	567 (53)	Patients	567 / 23.077	2.5
	55–65 years	207 (20)	Patients	207 / 11.601	1.8
GP’s sex	Female	34 (61)	GPs	694 / 30.880	2.2
	Male	22 (39)	GPs	372 / 20.542	1.8
GP age	< 50 years	23 (41)	GPs	487 / 20.580	2.4
	≥ 50 years	33 (59)	GPs	579 / 30.842	1.9
Practice size	1–2 GPs	13 (39)	Practices	352 / 17.186	2.0
	3+ GPs	20 (61)	Practices	714 / 34.236	2.1
Patients per GP	<1600	18 (55)	Practices	631 / 24.295	2.6
	≥1600	15 (45)	Practices	435 / 27.127	1.6
Household Income	≤60,000 €	7 (30)	Postal districts	482 / 23.228	2.1
	>60,000 €	16 (70)	Postal districts	584 / 28.194	2.1
Unemployment	≤3	5 (42)	Municipalities	301 / 16.536	1.8
	>3	7 (58)	Municipalities	765 / 34.886	2.2
Higher education	≤20	5 (42)	Municipalities	124 / 7.493	1.7
	>20	7 (58)	Municipalities	942 / 43.929	2.1
Urbanisation	≤75	5 (42)	Municipalities	284 / 11.909	2.4
	>75	7 (58)	Municipalities	782 / 39.513	2.0

The prevalence is the proportion (n) of patients aged 18-65 years with a consultation for psychological stress in the past 6 months based on a systematic search in EMRs and subsequent GP assessment. N was estimated from the total number of listed patients. We assumed that the sex and age composition of listed patients was similar to that of the local municipality population and that patients were equally distributed between participating and non-participating GPs. ^a^Two patients had no record of sex.

Multivariate analyses showed that several characteristics of patient, GP and local area were associated with psychological stress. Adjustments did generally not change the estimates considerably, except for area-related factors (Table 3). In the fully adjusted Model 4, a high risk of having a GP consultation for psychological stress was associated with: female sex, age 35–54 years, being listed with a female GP, being listed with a GP aged ≤50 years, being listed in a practice with one or two GPs, being listed in a practice with less than 1600 patients per GP, and living in an area with high education level and low population density. Living in an area with high income and high unemployment was not independent factors that were significantly associated with higher risk after mutual adjustments (Table 3).

**Table 3.** Patient-, GP- and area-related factors associated with having had a GP consultation for psychological stress in the six-months period.

Patient, GP and area characteristics		Model 1	Model 2	Model 3	Model 4
		OR (95% CI)	OR (95% CI)	OR (95% CI)	OR (95% CI)
Patient’s sex	Female	1	1	1	1
	Male	0.43 (0.35–0.52)	0.42 (0.34–0.51)	0.42 (0.34–0.51)	0.42 (0.34–0.51)
Patient’s age	18–34 years	1	1	1	1
	35–54 years	1.40 (1.16–1.69) 3.4)	1.39 (1.14–1.70)	1.39 (1.14–1.70)	1.38 (1.14–1.68)
	55–65 years	0.99 (0.78–1.27)	0.96 (0.76–1.23)	0.97 (0.76–1.23)	0.96 (0.76–1.21)
GP’s sex	Female	1	1		1
	Male	0.76 (0.55–1.04)	0.76 (0.59–0.99)		0.72 (0.56–0.92)
GP’s age	< 50 years	1	1		1
	≥ 50 years	0.77 (0.58–1.02)	0.80 (0.62–1.04)		0.73 (0.56–0.95)
Practice size	1–2 GPs	1	1		1
	3+ GPs	1.01 (0.73–1.40)	0.79 (0.58–1.06)		0.66 (0.46–0.95)
Patients per GP	< 1600	1	1		1
	≥ 1600-	0.61 (0.46–0.81)	0.59 (0.45–0.76)		0.64 (0.49–0.84)
Area, househ. income	≤ 60,000 €	1		1	1
	> 60,000 €	1.06 (0.78–1.45)		0.92 (0.69–1.22)	1.00 (0.76–1.32)
Area, unemployment	≤ 3%	1		1	1
	> 3%	1.27 (0.94–1.72)		1.11 (0.84–1.46)	0.94 (0.77–1.16)
Area, higher education	≤ 20%	1		1	1
	> 20%	1.39 (0.96–2.00)		1.55 (1.09–2.19)	1.74 (1.06–2.87)
Area, urbanisation	≤ 75%	1		1	1
	> 75%	0.90 (0.64–1.26)		0.70 (0.52–0.95)	0.67 (0.51-0.89)

All models are logistic regressions with robust estimates accounting for patient clustering with 56 different GPs. Model 1 is crude. Model 2 includes practice characteristics. Model 3 includes area characteristics. Model 4 includes both practice and area characteristics. The age- and sex-specific numbers of patients listed with the practices were estimated from the reported total number of listed patients and the age and sex composition in the GP´s municipality.

The majority (58%) of patients identified with psychological stress also had a psychiatric diagnosis, most often depression or anxiety.

Our subgroup analysis of psychological stress among patients listed with clinics in which all GPs participated showed a prevalence of 2.0%. Five clinics did not report a number of patients identified on the basis of the EMR search procedure, and they may not have used it. The prevalence was 2.2% when excluding these clinics.

## Discussion

### Main findings

The principal finding of the study is that about two per cent of working-age persons contacted the GP with psychological stress during six months and that the risk of having such a contact was positively associated with female sex and age 35–54 years. Exploratory analyses indicated that also GP characteristics, high level of education and low population density may be associated with higher prevalence of psychological stress.

### Comparison with existing literature

The 2% prevalence of psychological stress requiring health care in general practice corresponds to the findings in Danish national population surveys based on self-report questionnaires. These surveys found that about 2% of men and 3% of women have felt nervous or stressed very often in the past month (the questionnaires’ highest category out of five) [18–20].

The European Survey of Enterprises on New and Emerging Risks included 36 countries and ranged the Danish labour market in the high end regarding all psychosocial risks [21]. Nevertheless, the prevalence of psychological stress in Denmark has been reported to be similar to that in other countries in northern Europe [22].

The finding that psychological stress is most prevalent in the middle of working-age life agrees with a Danish population study conducted in year 2000 and the recent Danish National Working Environment Survey [20,23]. However, other Danish surveys from 2005 and 2013 found the highest levels of psychological stress in the youngest working-age group (16-24 years) and a gradual decrease in stress prevalence throughout working-age life [24,25]. The study methods differ in that the first-mentioned studies asked how often the respondent had felt stressed in daily/working life, whereas the latter studies used the Perceived Stress Scale (PSS) [26]. In the first-mentioned studies, psychological stress was associated with high job position, high education and among men also with high household income. In contrast, the latter studies found high levels of psychological stress to be associated with unemployment, low income and low education. One explanation for why the two methods identify persons with psychological stress differently may be that only the PSS includes the patient’s perception of control and predictability in the assessment of stress. Persons with high resources may report to have a stressful life, but they may also be more in control of it [27]. Unemployed persons or persons who are low in the working hierarchy are more likely to perceive their life as unpredictable and uncontrollable.

It is well known that both specialists and lay people differ substantially in their definitions and perceptions of stress [25,26]. However, it has not previously been shown that GP characteristics are associated with the frequency of psychological stress in the listed patients. As sick leave due to psychological stress is estimated to be among the largest disease-related costs in international society, it could be important to explore systematic differences among GPs in terms of stress assessment [21]. GPs are expected to agree on diagnoses and treatment, especially regarding diseases with major consequences for patients and society.

It has been shown that city living is stressful [28]. Our contrary finding that high prevalence of stress was associated with low urbanisation may be a coincidence as we only had 12 municipality urbanisation percentages in the study (Table 2) or may be a local, i.e. Danish, phenomenon.

### Strengths and limitations

The major strengths of this study include the high number of GP-listed patients who constitute the study cohort. Patients with severe psychological stress need to see the GP, e.g. for sick notes, evaluation of symptoms, treatment or referral to another healthcare professional; high completeness of data may, therefore, be assumed. Some patients may have contacted the GP because of psychological stress without being included in the study. However, these patients are expected to be few as the GPs were assisted by the electronic search on diagnostic codes. Coding of mental conditions and disorders has been mandatory for GPs in Denmark since 2014. Additionally, the GPs were incentivised to make thorough recordings because they needed to work with them at the seminar. Precise data on relevant area characteristics were available to analyse variations in the prevalence estimate. In the statistical analyses, robust estimates were used to counteract patient clustering caused by relatively few GPs in even fewer municipalities and postal districts.

A major limitation of the study is the risk of selection bias at the level of the GPs where only 7.3% of eligible GPs chose to participate. GPs with high interest in psychological stress may have been more prone to volunteer for the study. This group of GPs may also treat more of their patients for psychological stress, which might bias the study towards overestimating the prevalence. However, the prevalence estimate was almost the same, 2.0%, when including only clinics where all GPs participated compared to 2.1% including all clinics, and participating GPs were representative on major characteristics (Table 1). This suggests that our results may be cautiously extrapolated to settings comparable to Denmark.

The seminar itself did not affect the results of the audit as the audit was conducted retrospectively two months before the seminar.

Another major limitation of the study is that there is no consensus on diagnostic criteria and diagnosis codes to be used for stress. Consequently, we had no validation of the chosen diagnosis codes and their ability to capture and identify patients with psychological stress. Patients with stress could hide behind diagnoses that were not included in the search terms such as headache and back pain, and this could bias our prevalence estimate downwards. However, the computer search was just an assisting tool, and the five clinics that did not use it identified a similar frequency of patients with stress as the majority that used it.

The approach involving GPs to answer the questionnaire using electronic patient records is likely to have reduced recall bias by assisting the GPs’ memory on prescribed medication, referrals and sick notes. However, it may also have limited the specificity of the stress assessment because the patients were not involved in answering the questionnaire, and journal notes may not have fully described the mental health status of the patients. Some underestimation of the true prevalence of psychological stress may thus be suspected.

We assumed that the distribution of age and sex among listed patients were similar to that in the local municipality, and that patients were equally distributed between GPs in clinics with more than one GP. Thus, some clinics and GPs may have another patient mix than assumed in the calculations. However, we found virtually unchanged results when restricting our analyses to patients in clinics where all GPs participated, which indicated that this potential inaccuracy was of minor importance.

Some of the identified associations may be due to confounding. Female GPs have more female patients, and female sex is associated with higher risk of psychological stress. Likewise, GPs aged ≤50 years may have more patients aged 35–54 years who have the highest prevalence of psychological stress. Unfortunately, due to the design and the data availability of the study, we were unable to adjust for differences in the patient mix in the regression analyses. Hence, one possible explanation of the results could be that GPs meet similar patients, but they identify psychological stress differently. Another explanation could be that patients with psychological stress are more likely to choose GPs with certain characteristics. Furthermore, the associations between psychological stress in patients and GP characteristics could be caused by differences among GPs in the diagnostic coding or in the efforts of including patients in the study. GPs who are less keen on using diagnostic classification may tend to include fewer patients with psychological stress in the study although they identify and treat similar numbers of cases as other GPs.

## Conclusion

During a six-month period, about one in fifty of all patients in the working age contacted the GP for severe psychological stress. One in fifty may not sound as many, but this figure corresponds to almost one patient per week per GP and around 75,000 patients in Denmark during 6 months. Patients with stress often requires several consultations, including examinations of psychological and physical symptoms, talk therapy, introduction and adjustment of pharmaceutical treatment, sick notes and declarations to employer and municipality; psychological stress is associated with high use of both elective and acute services by the GP and in the hospitals [29, 30]. This reflects a substantial burden on society at large and on general practice in specific. There is a need to address this burden both at a societal level and in relation to future health service actions.

High risk of making contact to general practice with psychological stress is associated with female sex and age 35-54 years. It is also associated with the characteristics of the GP, low population density and high education level in the area where the patient lives. These findings need confirmation and further investigation in new studies. A special focus should be directed on exploring how management of psychological stress is associated with characteristics of the GP.
